# Oxidative Stress and Mitogen-Activated Protein Kinase Pathways Involved in Cadmium-Induced BRL 3A Cell Apoptosis

**DOI:** 10.1155/2013/516051

**Published:** 2013-03-21

**Authors:** Zhang Yiran, Jiang Chenyang, Wang Jiajing, Yuan Yan, Gu Jianhong, Bian Jianchun, Liu Xuezhong, Liu Zongping

**Affiliations:** College of Veterinary Medicine, Yangzhou University, Yangzhou 225009, Jiangsu, China

## Abstract

In this study, BRL 3A cells were treated with different Cd concentrations (0, 10, 20, and 40 **μ**mol/L) for 12 h and preincubated with or without N-acetyl-L-cysteine (NAC) (2 mmol/L) for 30 min, and cells were treated with Cd (0 and 20 **μ**mol/L), pretreated with p38 inhibitor (SB203580), JNK (c-Jun NH2-terminal kinases) inhibitor (SP600125), and extracellular signal-regulated kinase (ERK) inhibitor (U0126) for 30 min, and then treated with 20 **μ**mol/L Cd for 12 h. Cd decreased cell viability, SOD, and GSH-Px activity in a concentration-dependent manner. Increased MDA level, ROS generation, nuclear condensation, shrinkage, and fragmentation in cell morphology were inhibited by NAC. Cd-induced apoptosis was attenuated by pretreatment with SB203580, SP600125, and U0126. The results of western blot showed that NAC preincubation affected Cd-activated MAPK pathways, p38 and ERK phosphorylation. Cd treatment elevated the mRNA levels of Bax and decreased the mRNA levels of Bcl-2, respectively. The same effect was found in their protein expression levels. These results suggest that oxidative stress and MAPK pathways participate in Cd-induced apoptosis and that the balance between pro- and antiapoptotic genes (Bax and Bcl-2) is important in Cd-induced apoptosis.

## 1. Introduction

Cadmium (Cd) is one of the most toxic environmental and industrial pollutants. Acute or chronic exposure to this pollutant induces disturbances in several organs and tissues [1]. Occupational and environmental Cd pollution originates from mining, metallurgical industries, and manufacturers of nickel-Cd batteries, pigments, and plastic stabilizers. Important sources of human intoxication include cigarette smoke as well as food, water, and air contaminations. At the cellular level, Cd affects proliferation and differentiation. It also causes apoptosis. The International Agency for Research on Cancer has already classified Cd as a carcinogen [2].

Numerous studies revealed that Cd is a powerful inducer of oxidative stress [3], causing oxidative toxicity in broiler chicken, bone tissue, silver catfish, primary rat proximal tubular cells, and so on [4–9]. Cd decreases cell viability via a reactive oxygen species- (ROS-) mediated mechanism [10] and causes apoptosis through oxidative stress [8]. N-acetyl-L-cysteine (NAC) is a free radical scavenger used to determine the involvement of ROS in Cd-induced apoptosis and to suppress the renal proximal tubular damage caused by prolonged Cd exposure. Thus, oxidative stress has a critical function in Cd-induced toxicity [11]. However, the contributions of oxidative stress and apoptotic mechanisms to Cd-induced toxicity warrant further investigation.

In the pathways of oxidative stress-mediated apoptosis, mitogen-activated protein kinases (MAPKs) are given more attention in apoptosis. MAPKs, including extracellular signal-regulated kinases (ERKs), stress-activated protein kinases (c-Jun NH2-terminal kinases or JNK), and p38 MAPK, belong to a family of ser/thr protein kinases that mediate numerous complex cellular programs, such as cell proliferation, differentiation, and cell death in response to different stimuli [12–14]. ERK, which is activated by growth factors, is important for cell survival such as proliferation, differentiation, and development. By contrast, JNK and p38 MAPK are involved in apoptosis by promoting cell death rate [15, 16]. Several studies demonstrated that Cd activates MAPKs in neuronal cells [17, 18], immature hippocampus [19], and human retinal pigment epithelial cells [20]. Cd activates p38 MAPK and JNK in C6 rat glioma cells [21]. Yang et al. [22] suggested that Cd induces the apoptosis of the anterior pituitary both *in vivo* and *in vitro*. ERK and p38 MAPK pathways were found to be involved in the aforementioned processes. Similarly, Haase et al. [23] reported that monocytes/macrophages treated with Cd stimulate ERK and p38 MAPK phosphorylation. U-937 promonocytic cells treated with 200 *μ*mol/L CdCl_2_ for 2 h induce apoptosis, rapid p38 MAPK phosphorylation, and late ERK phosphorylation [24]. These reports suggest that the members of the MAPK family are activated by Cd exposure according to cell type, Cd concentration, and Cd exposure duration. The possible function of MAPK activation in Cd-induced apoptosis was determined using MAPK inhibitors, such as p38 (SB203580), ERK (U0126), and JNK (SP600125). However, the mechanism by which Cd activates the family members of MAPKs in BRL 3A cell line and whether Cd targets other signaling pathways responsible for BRL 3A cell survival remain unknown.

The protein and mRNA expression levels of Bcl-2 family members, such as Bax and Bcl-2, were investigated in Cd-induced apoptosis. Apoptotic-related proteins are members of the Bcl-2 family. These proteins regulate mitochondrial outer membrane permeabilization and can be either proapoptotic (Bax, Bak, and Bad) or antiapoptotic (Bcl-2, Bcl-xL, and Bcl-w). Bax regulates the mitochondrial pathway by triggering the release of apoptotic factors, such as cytochrome c (cyt c), from the mitochondria subsequent to the death signal [25]. Bcl-2 is an anti-apoptotic protein that regulates cell apoptosis by controlling mitochondrial membrane permeability and inhibits caspase activity either by preventing the release of cyt c from the mitochondria into the cytosol or by binding to the apoptosis-activating factor damage [26, 27]. Given the importance of Bcl-2 family members in apoptosis, the regulation of Bax and Bcl-2 in Cd-induced apoptosis is necessary to be determined.

Based on these considerations, this study investigated whether Cd can induce apoptosis. The effects of Cd on BRL 3A cells and the possible functions of oxidative stress, MAPK pathways, and Bcl-2 family members on Cd hepatotoxicity were also determined. We used BRL 3A cells to observe the effect of Cd and to explore the possible function of oxidative stress, MAPK pathways, and Bcl-2 family members on apoptosis induced by different concentrations of Cd and NAC. 

## 2. Materials and Methods

### 2.1. Materials

Cadmium acetate (CdAC_2_), 2-,7-dichlorofluorescein diacetate (DCFH-DA), penicillin, streptomycin, and Hoechst 33258 kit were purchased from Sigma-Aldrich (St. Louis, MO, USA). Fluorescein isothiocyanate (FITC) Annexin V apoptosis detection kits were purchased from BD Biosciences Pharmingen (San Diego, CA, USA). Dulbecco's modified Eagle's medium (DMEM) and fetal bovine serum (FBS) were obtained from Gibco Laboratories (Grand Island, NY, USA). 3-(4,5-Dimethylthiazol-2-yl)-2,5-diphenyl-tetrazolium bromide (MTT) and trypsin were from Amresco Inc. (Solon, OH, USA). Malondialdehyde (MDA), superoxide dismutase (SOD), and glutathione peroxidase (GSH-Px) kits were purchased from Jiancheng Bioengineering Institute (Nanjing, China). A bicinchoninic acid (BCA) protein assay kit was provided by Beyotime Institute of Biotechnology (Jiangsu, China). Antibodies antirat JNK, P-JNK, ERK, P-ERK, p38, P-p38, Bax, Bcl-2, *β*-actin, and horseradish peroxidase- (HRP-) conjugated goat antirabbit IgG were obtained from Cell Signaling Technology, Inc. (Danvers, MA, USA). Radio-immunoprecipitation assay (RIPA) lysis buffer was from Solarbio China, Inc. (Beijing, China). Kodak X-ray film was purchased from Eastman Kodak Co. (Rochester, NY, USA). Cell culture plates were obtained from Corning Inc. (Corning, NY, USA). PrimerScript RT reagent Kit and SYBR Premix Ex Taq were from TaKaRa Bio Inc. (Shiga, Japan). Nitrocellulose (NC) filter membrane was from Pall Gelman Sciences (Port Washington, NY, USA). Enhanced chemiluminescence (ECL) detection kit was from ECL Millipore Ltd. (Burlington, MA, USA). Other reagents used were available locally and of analytical grade.

### 2.2. Cell Culture and Treatments

BRL 3A-immortalized rat hepatocytes were used between passages 10 and 20 (Cell of Chinese Academy of Science, Shanghai, China). BRL 3A cells were cultured in DMEM culture medium (Gibco, USA) supplemented with 100 U/mL penicillin, 100 *μ*g/mL streptomycin, and 10% FBS. The cells were incubated at 37°C under a humid 5% CO_2_ atmosphere (Thermo, USA).

BRL 3A cells were seeded at a density of 2 × 10^5^ cells/mL in 6- or 96-well plates. CdAC2 was dissolved in distilled-deionized water as stock solution and diluted with nonserum culture medium to different concentrations before being added to the cell culture.

### 2.3. Cell Viability Assay

BRL 3A cells were treated with 0, 10, 20, and 40 *μ*mol/L Cd for 12 h. In the other two experiments, the cells were preincubated with 2 mmol/L NAC for 30 min and then incubated with 20 *μ*mol/L Cd for 12 h. Cell viability was evaluated by MTT assay. After the incubation period, the cells were incubated with MTT at a final concentration of 0.5 mg/mL for 4 h at 37°C prior to discarding the medium. Then, 150 *μ*L of DMSO was added to each well, and the plate was stirred thoroughly for 10 min on a shaker. The optical density (OD) of each well was measured at 490 nm with a sunrise-basic ELISA Reader (Tecan, Austria). Cell viability was expressed as the proportion of OD to the control.

### 2.4. Cell Morphological Analysis

BRL 3A cells were treated with 0, 10, 20, and 40 *μ*mol/L Cd for 12 h. In the other two experiments, the cells were incubated with 2 mmol/L NAC for 12 h and pre-incubated with 2 mmol/L NAC for 30 min, followed by incubation with 20 *μ*mol/L Cd for 12 h. After the treatment, images were taken with an inverted phase-contrast microscope (Leica, Germany) equipped with a Quick Imaging system.

### 2.5. Hoechst 33258 Staining

Nuclear morphology was analyzed using Hoechst 33258. BRL 3A cells were treated with 0, 10, 20, and 40 *μ*mol/L Cd for 12 h. In the other two experiments, the cells were incubated with 2 mmol/L NAC for 12 h and pre-incubated with 2 mmol/L NAC for 30 min, followed by incubation with 20 *μ*mol/L Cd for 12 h. The culture medium was removed after the treatment. The cells were washed twice with phosphate-buffered saline (PBS) and fixed in 4% formaldehyde at 4°C for 10 min. The fixed cells were washed, stained with 5 *μ*g/mL Hoechst 33258 at room temperature for 15 min in the dark, and then washed twice with PBS. Cell nuclear morphology was observed under a camera-equipped fluorescence light microscope using the filter of 450 nm to 490 nm. 

### 2.6. Determination of Apoptosis

BRL 3A cells were seeded into six-well plates. Apoptosis was tested using an apoptosis detection kit according to the manufacturer's instructions. BRL 3A cells were treated with 0 and 20 *μ*mol/L Cd for 12 h. In the other three experiments, the cells were pre-incubated with 10 *μ*mol/L SB203580, SP600125, and U0126 for 30 min, followed by incubation with 20 *μ*mol/L Cd for 12 h. After treatment, BRL 3A cells were collected and suspended in 100 *μ*L of binding buffer containing 5 *μ*L of FITC Annexin V and 5 *μ*L of propidium iodide (PI) dye solution. After incubation in the dark at 25°C for 15 min, 400 *μ*L of binding buffer was added. Then, the cells were analyzed by a FACSAria flow cytometer (Becton-Dickinson, San Jose, CA, USA) at excitation and emission wavelengths of 488 and 605 nm, respectively. A minimum of 10,000 cells per sample were registered. Quadrants were positioned on Annexin V/PI dot plots. Living (Annexin V−/PI−), early apoptotic (Annexin V+/PI−), late apoptotic (Annexin V+/PI+), and necrotic (Annexin V−/PI+) cells were distinguished. Therefore, the total apoptotic proportion included the percentage of cells with fluorescence Annexin V+/PI− and Annexin V+/PI+ [28].

Each independent experiment needed to set another three samples: unstained cells, FITC Annexin V only, and PI only. Each experiment was repeated at least three times.

### 2.7. ROS Determination 

The intracellular ROS levels were measured using the stable nonfluorescent molecule DCFH-DA. This molecule passively diffuses into cells, where the acetate can be cleaved by intracellular esterases to produce a polar diol that is retained well within the cells. Relative ROS production was expressed as a change in fluorescence compared with the fluorescence of the corresponding control. 

BRL 3A cells were treated with 0, 10, 20, and 40 *μ*mol/L Cd for 12 h. In the other two experiments, the cells were incubated with 2 mmol/L NAC for 12 h and pre-incubated with 2 mmol/L NAC for 30 min, followed by incubation with 20 *μ*mol/L Cd for 12 h. After the treatment, the cells were collected, incubated with 20 *μ*mol/L DCFH-DA at 37°C for 20 min in the dark, and then washed twice with PBS. The cells were analyzed in a FACSAria flow cytometer (Becton-Dickinson, USA) at excitation and emission wavelengths of 488 and 525 nm, respectively. 

### 2.8. Measurement of SOD Activity, GSH-Px Activity, and MDA Level 

As a breakdown product of the oxidative degradation of cell membrane lipids, MDA is generally considered as an indicator of lipid peroxidation. Lipid peroxidation was evaluated by measuring MDA concentrations through spectrophotometry of the color produced during the reaction of thiobarbituric acid with MDA. MDA concentrations expressed in nmol/mg protein were calculated from the absorbance of thiobarbituric acid reactive substances at 532 nm. SOD is a superoxide scavenger. Total SOD activity was determined from the inhibition rate of the superoxide radical-dependent cyt c reduction. In the assay, the xanthine-xanthine oxidase system was used as the source of superoxide ions, and the absorbance was determined at 550 nm. The values were expressed as U/mg protein. GSH-Px activity was assessed according to the kit's instruction via the reaction H_2_O_2_ + 2GSH → 2H_2_O + GSSG (oxidized glutathione). The absorbance was determined at 412 nm, and the enzyme activity was expressed as U/mg protein [28, 29].

BRL 3A cells were treated with 0, 10, 20, and 40 *μ*mol/L Cd for 12 h. In the other two experiments, the cells were incubated with 2 mmol/L NAC for 12 h and pre-incubated with 2 mmol/L NAC for 30 min, followed by incubation with 20 *μ*mol/L Cd for 12 h. The treated cells were pelleted and lysed in 200 *μ*L of cell lysis solution (containing 0.1 M Tris-HCl and 0.1% Triton-100) to evaluate lipid peroxidation following the protocol of SOD, GSH-Px, and MDA assay kits.

### 2.9. Western Blot Analysis

BRL 3A cells were treated with 0, 10, 20, and 40 *μ*mol/L Cd for 12 h. In the other two experiments, the cells were incubated with 2 mmol/L NAC for 12 h and pre-incubated with 2 mmol/L NAC for 30 min, followed by incubation with 20 *μ*mol/L Cd for 12 h (only in the ERK- and P38-related groups). After the treatment, the cells were washed twice with cold PBS, extracted into RIPA lysis buffer on ice for 30 min, and then sonicated at 3 W for 15 s. The cell lysates were centrifuged at 12,000 g for 10 min at 4°C. The protein content was determined using a BCA protein assay kit. Lysate aliquots were diluted with 6× sodium dodecyl sulfate (SDS) sample buffer and boiled for 10 min. A total of 30 *μ*g of protein from each treatment was separated by 12% SDS-polyacrylamide gel and then electrophoretically transferred onto NC membranes (Poll, USA). After being blocked at room temperature for 2 h with 5% nonfat milk in TBS with 0.1% Tween-20 (TBST), the membranes were incubated overnight at 4°C with the corresponding primary antibodies: rabbit anti-rat antibody (JNK, P-JNK, ERK, P-ERK, p38, P-p38, Bax, and Bcl-2) in 1 : 1000 and rabbit anti-rat *β*-actin antibody in 1 : 5000. After being washed with TBST (6 × 5 min), the membranes were incubated with HRP-conjugated goat anti-rabbit IgG (at a dilution of 1 : 5000) at room temperature for 2 h. After additional washes, the membranes were visualized using an ECL detection kit according to the manufacturer's instructions and then exposed to X-ray film. The volumes of the bands were determined by standard scanning densitometry with normalization of densitometry measures to *β*-actin.

### 2.10. Quantitative Real-Time Polymerase Chain Reaction (PCR) of Bax and Bcl-2

 BRL 3A cells were treated with 0, 10, 20, and 40 *μ*mol/L Cd for 12 h. After the treatment, RNA was extracted from BRL 3A cells using the AXYPrep multisource total RNA miniprep kit (Axygen, USA) according to the manufacturer's instructions. The OD ratios (OD260/OD280) of the samples were assessed between 1.8 and 2.0. For cDNA synthesis, 900 ng of total RNA was reverse transcribed to complementary DNA using a PrimerScript RT reagent kit with a gDNA eraser. After the RT reaction, each sample was conducted in triplicate, and each reaction mixture was prepared using the SYBR Premix Ex Taq in a total volume of 20 *μ*L. In a 96-well plate, cDNA fragments of Bax, Bcl-2, and *β*-actin were amplified separately by PCR in triplicate using an ABI PRISM7500 Sequence Detection System (Applied Biosystems, USA). The reaction conditions were as follows: 95°C for 2 min; 95°C for 5 s; 40 cycles of 95°C for 5 s, 59°C for 34 s, and 95°C for 15 s; 60°C for 1 min; and 95°C for 15 s. Relative quantification of gene expression within each reaction was calculated with the 2^−ΔΔCt^ method according to the manufacturer's instructions (ABI PRISM 7500 Sequence Detection System, Applied Biosystems, USA). 

The primer sequences (Table 1) were designed according to cDNA sequences from Gene Bank. All primers were synthesized by Invitrogen China, Inc. (Shanghai, China).

### 2.11. Statistical Analysis

Results were represented statistically as means ± SD. Significance was assessed by one-way ANOVA following appropriate transformation to normalized data and equalized variance where necessary. Statistical analysis was performed using SPSS statistics 17.0 (SPSS Inc., USA); *P* < 0.05 and *P* < 0.01 were considered to indicate significance and high significance, respectively. All assays were performed in triplicate.

## 3. Results

### 3.1. Cell Viability 

**Figure 1:**
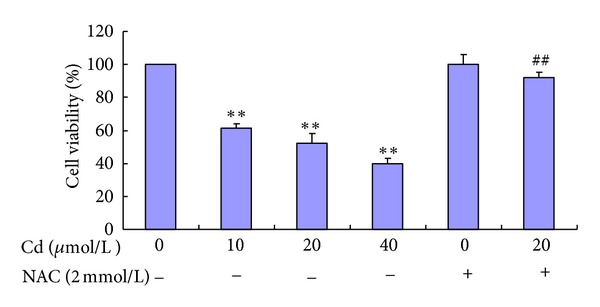
Cell viability of BRL 3A cells treated with Cd and NAC. Cells were treated with 0 *μ*mol/L to 40 *μ*mol/L Cd and pre-treated with or without 2 mmol/L NAC for 12 h. Data are presented as mean ± SD of three independent experiments performed in triplicate. Significant difference: *P < 0.05, **P < 0.01, compared with the control. ^#^ *P* < 0.05, ^##^ *P* < 0.01, compared with the 20 *μ*mol/L Cd group.

To determine the appropriate concentration of Cd for the mechanism studies, we measured the effect of Cd exposure on cell viability. As shown in Figure 1, Cd (0 *μ*mol/L to 40 *μ*mol/L) decreased cell viability in a concentration-dependent manner. The cell viability of the 20 *μ*mol/L Cd group was approximately 50% of that of the control. Therefore, 20 *μ*mol/L of Cd was used in the experiments of inhibitory effects. NAC (2 mmol/L) alone did not obviously alter cell viability compared with the control. However, preincubation with 2 mmol/L NAC for 30 min attenuated the reduction in cell viability induced by 20 *μ*mol/L Cd compared with the 20 *μ*mol/L Cd group.

### 3.2. Effects of Cd on Cell Morphology

Phase-contrast microscopic observations after exposure to increasing Cd concentrations (10 *μ*mol/L to 40 *μ*mol/L) revealed morphological changes showing cytoplasmic shrinkage, rounding, and loss of cell integrity. NAC (2 mmol/L) alone had no significant effect on cell morphology compared with the control. Pre-incubation with 2 mmol/L NAC for 30 min could attenuate cytotoxicity to maintain cell integrity induced by 20 *μ*mol/L Cd compared with the 20 *μ*mol/L Cd group.

**Figure 2:**
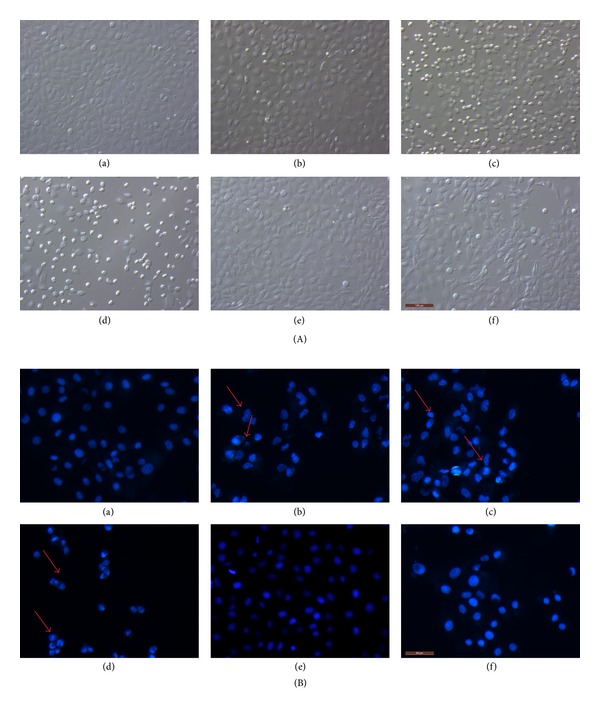
Cd and NAC induce morphological changes in BRL 3A cells. Cultured cells were exposed to 0, 10, 20, and 40 *μ*mol/L Cd for 12 h (a, b, c, and d). In the other experiment, the cells were pre-incubated with 2 mmol/L NAC for 30 min and incubated with 20 *μ*mol/L Cd or only incubated with 2 mmol/L NAC for 12 h (f and e). (A) After the treatment, images were taken with an Olympus inverted phase-contrast microscope. (B) Thereafter, the cells were fixed, stained with Hoechst 33258, and then observed under a fluorescence microscope. Arrows indicate morphological changes (blebbing cells, chromatin condensation, or fragmentation) in the nuclei of BRL 3A cells. Scale bar: 100 *μ*m.

Following detection of apoptosis-related proteins and genes, BRL 3A cell apoptosis was observed after Hoechst 33258 staining. Significant morphological changes were observed in the cells exposed to 10 *μ*mol/L to 40 *μ*mol/L Cd for 12 h. The cells of the control group appeared normal with round and homogenous nuclei, whereas those treated with 10 *μ*mol/L to 40 *μ*mol/L Cd exhibited typical apoptotic features, such as plasma membrane blebbing, cell shrinkage, fragmentation, and nuclear chromatin condensation. The fluorescence intensity of cell staining with Hoechst 33258 indicated that the untreated cells displayed evenly dispersed chromatin structures. However, the cells treated with NAC (2 mmol/L) alone showed no significant changes compared with the control cells. Pre-incubation with 2 mmol/L NAC could attenuate changes, such as abnormal nuclear contents (Figure 2).

### 3.3. Effects of Cd on Cell Apoptosis

**Figure 3:**
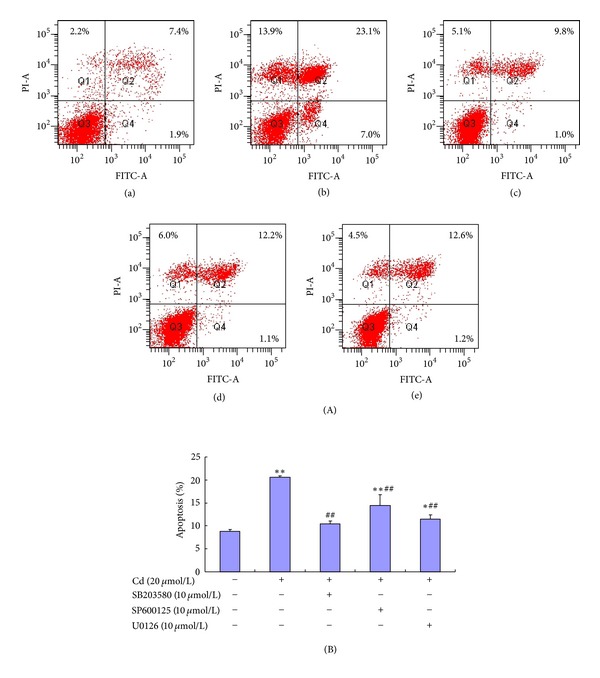
Flow cytometric analysis of BRL 3A cells after treatment with Cd and MAKP inhibitors. Cellular apoptosis was tested using an apoptosis detection kit. (A) BRL 3A cells were treated with 0 and 20 *μ*mol/L Cd for 12 h (a and b). In the other three experiments, the cells were pre-incubated with 10 *μ*mol/L SB203580, SP600125, and U0126 for 30 min, followed by incubation with 20 *μ*mol/L Cd for 12 h (c, d, and e). (B) The apoptotic percentage shows that 10 *μ*mol/L p38 inhibitor (SB203580), JNK inhibitor (SP600125), and ERK inhibitor (U0126) can reduce cell apoptosis significantly. Data are presented as mean ± SD of three independent experiments performed in triplicate. Significant difference: *P < 0.05, **P < 0.01, compared with the control. ^#^ *P* < 0.05, ^##^ *P* < 0.01, compared with the 20 *μ*mol/L Cd group.

 The involvement of MAPK signaling in Cd-induced apoptosis was investigated. Flow cytometry was used to distinguish the effects of MAPK inhibitors in apoptosis and necrosis after double staining with Annexin V-FITC and PI. Pre-incubation with 10 *μ*mol/L SB203580, SP600125, and U0126 for 30 min before treatment with 20 *μ*mol/L Cd significantly decreased cell apoptotic rates to 10.47%, 14.43%, and 11.47%, respectively, in BRL 3A cells compared with the corresponding rate of 20.6% in the 20 *μ*mol/L Cd control group (Figure 3).

### 3.4. Effects of Cd on ROS Generation

**Figure 4:**
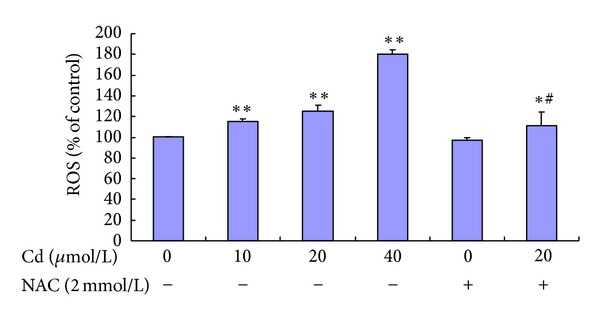
Effect of Cd and NAC on ROS generation of BRL 3A cells. Cultured cells were exposed to 0 *μ*mol/L to 40 *μ*mol/L Cd and pre-treated with or without 2 mmol/L NAC for 12 h. Data are presented as mean ± SD of three independent experiments performed in triplicate. Significant difference: *P < 0.05, **P < 0.01, compared with the control. ^#^ *P* < 0.05, ^##^ *P* < 0.01, compared with the 20 *μ*mol/L Cd group.

ROS generation was expressed as the measured fluorescence intensity in analyzed cells. Representative results of ROS are shown in Figure 4. Cd (10 *μ*mol/L to 40 *μ*mol/L) increased the level of ROS production in a concentration-dependent manner, whereas pre-incubation with 2 mmol/L NAC significantly reduced ROS generation induced by 20 *μ*mol/L Cd.

### 3.5. Effects of Cd on SOD Activity, GSH-Px Activity, and MDA Level

**Figure 5:**
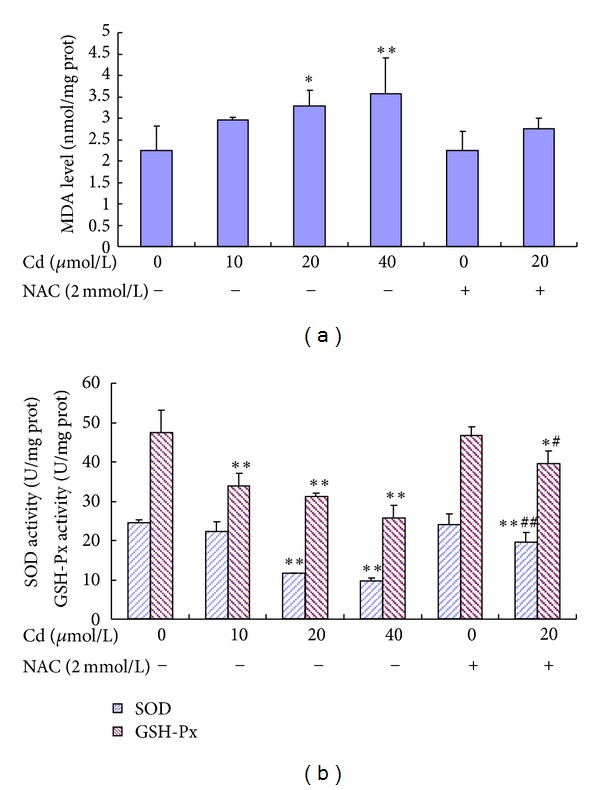
Effect of Cd and NAC on MDA level (A), SOD activity, and GSH-Px activity (B) in BRL 3A cells. Cells were treated with 0 *μ*mol/L to 40 *μ*mol/L Cd and pre-treated with or without 2 mmol/L NAC for 12 h. Data are presented as mean ± SD of three independent experiments performed in triplicate. Significant difference: *P < 0.05, **P < 0.01, compared with the control. ^#^ *P* < 0.05, ^##^ *P* < 0.01, compared with the 20 *μ*mol/L Cd group.

To assess the intracellular oxidant and antioxidant status, SOD activity, GSH-Px activity, and MDA level were evaluated in BRL 3A cells (Figure 5). The MDA levels in the groups treated with 20 *μ*mol/L to 40 *μ*mol/L Cd were remarkably higher than those in the control group. Cd (20 *μ*mol/L to 40 *μ*mol/L) significantly decreased SOD activity. Compared with the 20 *μ*mol/L Cd group, SOD activity significantly increased in the group pre-incubated with 2 mmol/L NAC. The GSH-Px activity in the groups treated with 10 *μ*mol/L to 40 *μ*mol/L Cd was significantly lower than that in the control group. The GSH-Px activity in the cells pre-incubated with 2 mmol/L NAC was significantly higher than that in the cells treated with 20 *μ*mol/L Cd alone.

### 3.6. Effects of Cd on Protein Expression of p38, ERK, and JNK

Immunoblot analyses were performed with antibodies to determine the effects of Cd on the phosphorylation of MAPKs. The effects of Cd exposure on protein expression were also observed. Western blot analysis showed that the treatment of BRL 3A cells with Cd for 12 h caused some changes. Robust phosphorylation of p38 and JNK (1/2) was observed in the groups treated with 10 *μ*mol/L to 40 *μ*mol/L Cd, whereas decreased phosphorylation of ERK was found in the groups treated with 10 *μ*mol/L to 20 *μ*mol/L Cd. Meanwhile, 40 *μ*mol/L Cd did not significantly alter the phosphorylation of 44 kD ERK but significantly elevated the phosphorylation of 42 kD ERK. ERK phosphorylation could be increased and p38 phosphorylation could be inhibited by NAC pre-incubation compared with the 20 *μ*mol/L Cd group. 

**Figure 6:**
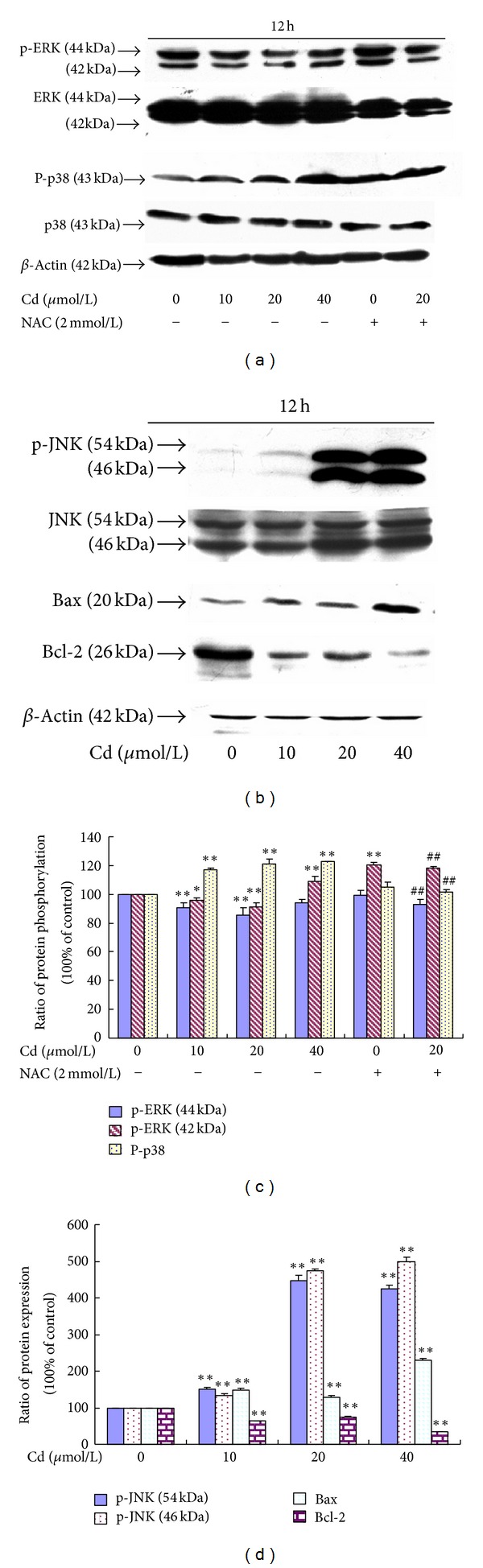
(a) Effect of Cd and NAC on ERK and p38 phosphorylation of BRL 3A cells. (b) Effect of Cd on the JNK phosphorylation and Bax and Bcl-2 protein expression levels of BRL 3A cells. (c) Quantitative analysis of the immunoreactive phosphorylated ERK and p38 proteins in treated BRL 3A cells. (d) Quantitative analysis of the immunoreactive phosphorylated JNK, Bax, and Bcl-2 proteins in Cd-treated BRL 3A cells. Each value is expressed as the phospho/total MAPK percentage of phosphorylation and the ratio of OD in Bax and Bcl-2 with respect to *β*-actin. Significant difference: *P < 0.05, **P < 0.01, compared with the control. ^#^ *P* < 0.05, ^##^ *P* < 0.01, compared with the 20 *μ*mol/L Cd group.

A significant increase in Bax protein level was found in the groups treated with 10 *μ*mol/L to 40 *μ*mol/L Cd. A significant reduction in Bcl-2 protein level was observed in the groups treated with 10 *μ*mol/L to 40 *μ*mol/L Cd. The data shown (Figure 6) are expressed as percentage of the control (considered as 100%).

### 3.7. Effects of Cd on the mRNA Levels of Bax and Bcl-2

**Figure 7:**
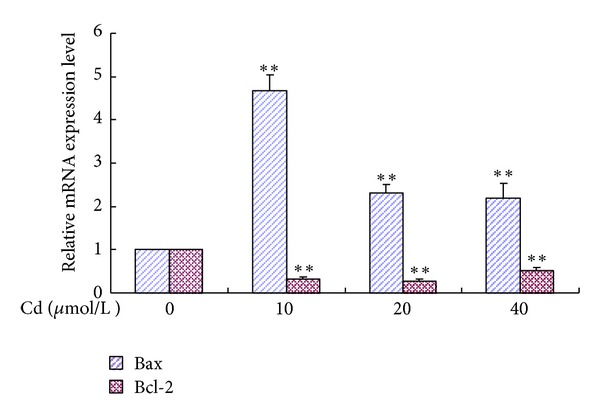
Relative quantification of Bax and Bcl-2 gene expression levels by real-time PCR in relation to *β*-actin. Cells were treated with 0 *μ*mol/L to 40 *μ*mol/L for 12 h. Data are presented as mean ± SD of three independent experiments performed in triplicate. Significant difference: *P < 0.05, **P < 0.01, compared with the control; ^#^ *P* < 0.05, ^##^ *P* < 0.01, compared with the 20 *μ*mol/L Cd group.

Transcriptional changes in Bax and Bcl-2 were observed in the Cd-treated BRL 3A cells. The Bax mRNA level was significantly elevated in the groups treated with 10 *μ*mol/L to 40 *μ*mol/L Cd, with the peak found in the 10 *μ*mol/L Cd group. The Bcl-2 mRNA level in the groups treated with 10 *μ*mol/L to 40 *μ*mol/L Cd significantly decreased.  Figure 7 shows the relative quantification of gene expression levels by real-time PCR in relation to *β*-actin.

## 4. Discussion

In the present study, Cd was demonstrated to be toxic to BRL 3A cells, resulting in decreased cell viability and increased oxidative stress. Furthermore, MAPK pathways and Bax gene family have critical functions in Cd-induced apoptosis. The antioxidant NAC attenuated most of these changes. Thus, ROS elevation is an early event in Cd-induced apoptosis. 

In this study, 10 *μ*mol/L to 40 *μ*mol/L Cd significantly decreased cellular viability. This finding may be attributed to apoptotic cell death. Chen et al. [17] reported that neuronal cell apoptosis is induced by 10 *μ*mol/L Cd, whereas Iryo [30] observed apoptosis in CCRF-CEM cells treated with 5 *μ*mol/L Cd. Exposure to 5 *μ*mol/L Cd decreases granulosa cell number and viability and causes chromatin condensation and DNA fragmentation [28]. Thus, Cd at doses higher than 5 *μ*mol/L is harmful to cells *in vitro* possibly because of apoptosis induction.

Apoptosis is a major mode of elimination of damaged cells in Cd toxicity and precedes necrosis [31]. In the present study, morphological observations and flow cytometric assessment showed that Cd induced the apoptosis of BRL 3A cells. This observation is compatible with the report of Coonse et al. [32], who proved the occurrence of apoptosis after Cd treatment of the human osteoblast-like cell line Saos-2. In addition, morphological and biochemical analyses revealed that Cd induces the apoptosis of murine fibroblasts [33].

The mechanisms of Cd toxicity have been suggested to interfere with cell adhesion and signaling, oxidative stress, apoptosis, genotoxicity, and cell cycle disturbance [34]. Although the overall effect of Cd on any cell or tissue is likely to be due to a synergism of several mechanisms, only one mechanism possibly dominates in a specific cell type [28]. In these studies, the toxic manifestations induced by Cd were associated with oxidative stresses, including lipid peroxidation and ROS production. Previous studies found that oxidative stress can be induced by Cd. Moreover, Cd-induced apoptosis is mediated by oxidative stress in LLC-PK1 [11]. Aydin et al. [31] demonstrated that Cd induces oxidative stress, resulting in oxidative deterioration of biological macromolecules. Cd possibly affects bone tissues through disorders in its oxidative/antioxidative balance, resulting in oxidative stress [5]. ROS reportedly possess important functions in the initiation of apoptosis. Bertin and averbeck [2] confirmed that Cd can provoke ROS generation. NAC is an antioxidant and ROS scavenger that can effectively block the Cd-induced activation of ERK, JNK, and p38 signaling network, prevent Cd-induced cell death, and significantly reduce Cd-induced toxicity in human lens epithelial cells and human retinal pigment epithelial cells [17, 20, 35]. These findings demonstrate the association between apoptosis and intracellular ROS. Similarly, Chen showed that Cd induces ROS generation, leading to apoptosis of PC12 and SH-SY5Y cells. Pretreatment with NAC scavenged Cd-induced ROS and prevented cell death, suggesting that Cd-induced apoptosis is caused by ROS generation. Thus, antioxidants can be exploited for the prevention of Cd-induced diseases [17]. The present study showed that Cd elevated ROS generation and NAC antagonized Cd-induced ROS. As ROS scavengers, SOD and GSH-Px were depleted. As a lipid peroxidation product, MDA accumulated in BRL 3A cells exposed to Cd. NAC elevated the activities of SOD and GSH-Px. The results of the present study are in accordance with previous reports, suggesting that oxidative stress has a major function in BRL 3A cells exposed to Cd. The toxic influence of Cd is most likely due to the formation of excess free radicals that cause oxidative stress, resulting in cell damage. Similarly, Cd can inhibit SOD and GSH-Px in human embryonic kidney cells, suggesting enhanced ROS levels [36]. Cd treatment significantly increased MDA level and decreased GSH-Px and SOD activities in granulosa cells from chicken ovarian follicles [28]. Cd exposure increases MDA content and reduces GSH-Px and SOD activities in the frontal cortex and hippocampus [37]. Moreover, exposure of yeast cells to Cd increases MDA level. By contrast, SOD and GSH-Px activities were also high in Cd-exposed cells [7]. The present study found that NAC cannot block MDA. These discrepancies may be dependent on cell type, stimulus, Cd concentration, and Cd exposure duration. 

MAPKs are important signal enzymes in controlling cell survival, proliferation, and differentiation. They are also involved in many facets of cellular regulation. ERK, which is currently believed to be activated by growth factors, is necessary for cell proliferation, differentiation, and development. By contrast, JNK and p38 are involved in apoptosis by promoting cell death rate [15, 16]. Previous studies demonstrated that the activation of MAPK pathways is responsible for Cd-induced apoptosis in various cells. Cd was reported to activate MAPKs in human retinal pigment epithelial cells [20] and human lens epithelial cells [35]. Several studies noted that Cd activates the MAPKs in neuronal cells [17, 18]. The present results showed that treating BRL 3A cells with 10 *μ*mol/L to 40 *μ*mol/L Cd for 12 h resulted in the robust phosphorylation of p38 and JNK (1/2). Meanwhile, p38 phosphorylation was inhibited by NAC preincubation compared with the 20 *μ*mol/L Cd group. The present findings indicated that Cd induced the apoptosis of BRL 3A cells at least partially by activating p38 and JNK (1/2) and that p38 phosphorylation can be inhibited by NAC pre-incubation. Thus, p38 and JNK (1/2) both participate in Cd-induced apoptosis. In addition, oxidative stress might lay upstream of p38, indicating a key function in its activation. Cd at 10 and 20 *μ*mol/L decreased the phosphorylation of ERK. Conversely, 40 *μ*mol/L Cd did not significantly alter the phosphorylation of 44 kD ERK but significantly elevated the phosphorylation of 42 kD ERK. ERK phosphorylation was increased by NAC pre-incubation, which is contradicting to previous findings. ERK is involved in the regulation of proliferation and apoptosis in several cells [38]. ERK has also been associated with two apparently opposing processes. The involvement of ERK in cell proliferation has been extensively described, as well as its function in postmitotic cells undergoing differentiation. Depending on the cell type and stimulus, ERK also acts as a negative regulator of cell proliferation and induces apoptosis when its activity is highly increased [38, 39]. Analysis of these apparent discrepancies led to a more precise understanding of the multiple functions and regulations of ERK [40]. The present results showed no significant change between the control and 40 *μ*mol/L Cd groups in the phosphorylation of 44 kD ERK. By contrast, the phosphorylation of 42 kD ERK increased significantly. These results suggest that low Cd concentrations result in an unconventional ERK phosphorylation, which in turn leads to apoptosis signaling. High Cd concentrations can activate ERK phosphorylation. This claim is in accordance with the theory that the final cellular outcome of activated ERK is dependent on the cell type, the stimulus that induces ERK, and the duration of ERK activation [41].

To investigate the relationship between MAPK phosphorylation and apoptosis, BRL 3A cells were pretreated with SB203580, SP600125, and U0126 for 30 min before treatment with 20 *μ*mol/L Cd. Pretreatment with these inhibitors significantly blocked Cd-induced apoptosis, indicating that p38, ERK, and JNK are involved in BRL 3A cells exposed to Cd and that MAPK pathways are the downstream pathways of oxidative stress in apoptosis. Similarly, in human promonocytic cells, the p38-specific inhibitor SB203580 can attenuate apoptosis [24]. In PC12 and SH-SY5Y cells, inhibition of ERK (U0126) and JNK (SP600125), but not p38 (SB203580), partially protects the cells from Cd-induced apoptosis. In CCRF-CEM cells, treatment with the ERK inhibitor U0126 suppresses Cd-induced ERK activation and apoptosis, whereas the inhibition of p38 activity with SB203580 cannot protect cells from apoptosis [30]. By contrast, SB202190 is a p38 inhibitor that decreases the cytotoxicity and apoptosis induced by high Cd concentrations [42]. In summary, some MAPK inhibitors suppress cell death and apoptosis depending on the concentrations of Cd and inhibitors. This finding indicates that JNK, ERK, and p38 independently participate in Cd-induced cell death and apoptosis. These results strongly suggest that MAPKs have different functions in Cd-exposed BRL 3A cells and that MAPK inhibitors can prevent Cd-induced toxicity, even though other signaling pathways are involved in the Cd-induced toxicity. Three major apoptosis pathways are involved in mammalian cells: mitochondria-, death receptor-, and endoplasmic reticulum-mediated apoptosis. Several studies showed cell apoptosis via mitochondria, death receptor, and endoplasmic reticulum pathways during Cd exposure. Coutant et al. [43] suggested that Cd-induced apoptosis can occur in the Boleth cell line through caspase-dependent and -independent pathways. Cd-induced apoptosis was investigated in LLC-PK1 cells via ROS- and mitochondria-linked signal pathways [11]. Endoplasmic reticulum (ER) stress signaling and mitochondrial pathways mediate Cd-induced testicular germ cell apoptosis [44]. Cd can induce apoptosis via the mitochondrial pathway in human embryonic kidney cells [36]. Apoptosis is usually controlled by the coeffects of different signal pathways rather than any single pathway. Thus, *in vitro* studies on the mitochondrial, death receptor, and ER pathways in Cd-exposed BRL 3A cells should be prioritized in the future.

The Bcl-2 family members were found to play important roles in regulating mitochondrial-mediated apoptosis. The Bcl-2 family is divided into two groups based on function. Members of the first group, such as Bcl-2 and Bcl-xL, have anti-apoptotic activity and protect cells from death. By contrast, Bax, Bad, and Bid, as members of the second group, show pro-apoptotic activity [45]. In addition, Bcl-2 was observed to promote cell survival by preserving the integrity of the external mitochondrial membrane, which prevents the release of cyt C from the mitochondria, inducing cell death [26]. Bax is a 21 kDa protein that promotes mitochondrial membrane permeability, leading to apoptotic cell death [46]. The present study showed an increase in Bax and a decrease in Bcl-2 in protein expression and mRNA level. Similarly, Zhou et al. [11] revealed that Bcl-2 protein expression can decrease significantly and Bax protein expression can increase as early as 12 h after exposure to Cd in LLC-PK1 cells. Cd induces apoptosis by provoking higher Bax expression and inhibiting Bcl-2 expression in granulosa cells from chicken ovarian follicles [28]. These results suggest that the up- or downregulation of Bax and Bcl-2 by Cd accounts for its pro- or anti-apoptotic effect on BRL 3A cells *in vitro*. 

## 5. Conclusion

Cd can enhance oxidative stress and induce MAPK pathway activation. Inhibition of p38, JNK, and ERK protected the cells from Cd-induced apoptosis. The apoptosis of BRL 3A cells was also associated with the Bcl-2 family. This study partly elucidates the hepatotoxic mechanism of* in vitro *exposure to Cd and offers opportunities for the development of therapeutic agents for Cd-induced hepatic diseases. 

## Figures and Tables

**Table 1:** The primers for real-time PCR.

Gene	Primers (5′-3′)
Bax	Sense: TTGTTACAGGGTTTCATCCAGG
	Antisense: GTGTCCACGTCAGCAATCATC
Bcl-2	Sense: GGGAGCGTCAACAGGGAG
	Antisense: AGCCAGGAGAAATCAAACAGA
*β*-Actin	Sense: CGTTGACATCCGTAAAGAC
	Antisense: TGGAAGGTGGAGTGAG
